# Association between metabolic dysfunction-associated steatotic liver disease and risk of urolithiasis: an updated systematic review and meta-analysis

**DOI:** 10.1007/s11739-024-03705-5

**Published:** 2024-07-11

**Authors:** Alessandro Mantovani, Riccardo Morandin, Veronica Fiorio, Maria Giovanna Lando, Salvatore Petta, Pietro Manuel Ferraro, Giovanni Targher

**Affiliations:** 1 Section of Endocrinology, Diabetes and Metabolism, Department of Medicine, University of Verona, Verona, Italy; 2 Section of Gastroenterology and Hepatology, PROMISE, University of Palermo, Palermo, Italy; 3 Section of Nephrology, Department of Medicine, University of Verona, Verona, Italy; 4 Department of Medicine, University of Verona, Verona, Italy; 5 Metabolic Diseases Research Unit, IRCCS Sacro Cuore, Don Calabria Hospital, Viale Luigi Rizzardi, 4, 37024 Negrar di Valpolicella (VR), Italy

**Keywords:** Nonalcoholic fatty liver disease, NAFLD, Metabolic dysfunction-associated steatotic liver disease, MASLD, Urolithiasis, Renal stones, Meta-analysis

## Abstract

Epidemiological studies have reported an association between metabolic dysfunction-associated steatotic liver disease (MASLD) and the risk of urolithiasis. However, the magnitude of the risk and whether this risk varies with the severity of MASLD remains uncertain. We performed a meta-analysis of observational studies to quantify the magnitude of the association between MASLD and urolithiasis. We systematically searched PubMed, Scopus, and Web of Science from database inception to March 31, 2024, using predefined keywords to identify relevant observational studies in which imaging methods or survey questionnaires diagnosed MASLD and urolithiasis. Meta-analysis was performed using random-effects modelling. We identified seven cross-sectional studies and one prospective cohort study with aggregate data on 248,936 adults from different countries. MASLD was significantly associated with an increased risk of prevalent urolithiasis (pooled random-effects odds ratio 1.87, 95% CI 1.34–2.60; *I*^2^ = 91%). This association remained significant in those studies whose results were adjusted for age, sex, ethnicity, obesity, diabetes, and other potential confounders. There was a positive graded association between the ultrasonographic severity of MASLD and urolithiasis. Meta-analysis of the single prospective cohort study showed that MAFLD was not associated with risk of developing incident urolithiasis (pooled random-effects hazard ratio 1.08, 95% CI 0.90–1.30), although a significant association was reported in men. Sensitivity analyses did not modify these findings. The funnel plot did not reveal any significant publication bias. This updated meta-analysis provides evidence for a significant association between MASLD and the presence of urolithiasis. Whether MASLD is associated with a higher risk of developing incident urolithiasis remains to be established.

**Supplementary Information:**

The online version contains supplementary material available at 10.1007/s11739-024-03705-5.

## Introduction

Non-alcoholic fatty liver disease (NAFLD), recently renamed as metabolic dysfunction-associated steatotic liver disease (MASLD), has emerged as a public health threat as it affects up to ~ 38% of the adult population worldwide [1–3], with its global prevalence rising in parallel with that of obesity and metabolic syndrome. An estimated ~ 50% of the worldwide adult population is forecasted to have MASLD by 2040 [2]. In 2023, using a multi-society Delphi process that involved 236 panelists from 56 countries, three large pan-national liver associations (i.e., the American Association for the Study of Liver Diseases, the European Association for the Study of the Liver, and the Latin American Association for the Study of the Liver) have endorsed the newly proposed change in nomenclature from NAFLD to MASLD [1]. Therefore, for sake of clarity, we have opted to use the term MASLD throughout the manuscript.

Beyond its liver health implications, a rapidly expanding body of evidence indicates that MASLD is a multisystem disease extending its adverse effects beyond the liver [4, 5], and has been associated with an increased risk of developing extrahepatic cancers (mainly gastrointestinal and gynecological cancers) [6] and cardiometabolic comorbidities, such as type 2 diabetes [7], adverse cardiovascular outcomes [8–10] and chronic kidney disease [11].

Among the least investigated extrahepatic complications related to MASLD, an ever-increasing number of observational studies have reported an increased prevalence of urolithiasis in adults with MASLD (as extensively discussed below). Urolithiasis has become one of the most common urinary system diseases worldwide, with an estimated prevalence ranging from ~ 1 to 20% in different regions across the globe [12]. Evidence also suggests that the prevalence of urolithiasis is on the rise globally due to changes in socio-economic conditions, climatic factors and dietary habits, as well as increasing rates of metabolic comorbidities (such as overweight/obesity, metabolic syndrome and type 2 diabetes) [12, 13]. Therefore, the increasing global burden of urolithiasis is expected to have substantial health, economic and social implications in the coming years.

To our knowledge, two previous small meta-analyses (published in 2018) have reported that the risk of urolithiasis in individuals with MASLD appeared to be higher than in those without MASLD [14, 15]. These meta-analyses did not address the question of whether the strength of any association between MASLD and urolithiasis was affected by severity of NAFLD. Notably, after the publication of these two small meta-analyses, it is important to note that new large observational studies have been published on this topic.

We have, therefore, carried out an updated systematic review and meta-analysis of observational studies to quantify the magnitude of the association of urolithiasis in adult individuals with MASLD. We also aimed to examine whether the severity of MASLD was associated with an increased risk of urolithiasis. Clarifying the magnitude of the risk of urolithiasis related to MASLD may favorably impact the development of primary prevention strategies for urolithiasis.

## Methods

### Registration of review protocol

The protocol of the systematic review was registered on the Open Science Framework (OSF) database (registration DOI: 10.17605/OSF.IO/JVGP2).

### Data sources and searches

This systematic review has been performed following the Preferred Reporting Items for Systematic Reviews and Meta-Analyses (PRISMA) and the Meta-analysis Of Observational Studies in Epidemiology (MOSE) guidelines [16, 17]. We systematically searched three large electronic databases (PubMed, Web of Science and Scopus) from database inception to March 31, 2024, to identify relevant observational studies examining the association between MASLD and the risk of urolithiasis. Search free text terms were "urolithiasis" OR "nephrolithiasis" OR "renal stones" OR “urinary calculi” AND “fatty liver” OR “non-alcoholic fatty liver disease” OR “NAFLD” OR “non-alcoholic steatohepatitis” OR “metabolic dysfunction-associated steatotic liver disease” OR “MASLD” OR “metabolic dysfunction-associated steatohepatitis” OR “metabolic dysfunction-associated fatty liver disease” OR “MAFLD”. Searches were restricted to human studies and English language studies. Subjects included in the meta-analysis were of either sex without age, race, or ethnicity restrictions.

### Study selection

Eligible studies were included in the meta-analysis if they met the following inclusion criteria: (1) observational (cross-sectional, case–control, or longitudinal) studies examining the risk of urolithiasis amongst adult (age ≥ 18 years) individuals with and without MASLD (or NAFLD); (2) studies that reported odds ratios (ORs) or hazard ratios (HRs) with 95% confidence intervals (95% CIs) values for the outcome of interest; (3) the diagnosis of MASLD (or NAFLD) was based on liver biopsy, imaging techniques or blood-based biomarkers/scores, in the absence of significant alcohol consumption (usually defined as < 20 g/day for women and < 30 g/day for men) or other competing causes of hepatic steatosis (e.g., viral hepatitis, iron overload and use of potentially hepatotoxic drugs); and (4) the diagnosis of urolithiasis was based on imaging techniques (ultrasonography or computed tomography) or a self-reported history.

The exclusion criteria of the meta-analysis were as follows: (1) congress abstracts, case reports, reviews, practice guidelines, commentaries or editorials; (2) studies in which the diagnosis of MASLD (or NAFLD) was based exclusively on serum liver enzyme concentrations; (3) studies which did not exclude individuals with significant alcohol consumption or other known causes of chronic liver disease; and 4) studies that included exclusively patient populations with MASLD (or NAFLD) or those with known or suspected urolithiasis.

### Data extraction and quality assessment

Data from studies eligible for the aggregate data meta-analysis were independently extracted by two investigators (AM and GT). Any disagreements between investigators about including eligible studies were resolved by consensus and a third investigator if needed (RM).

For each eligible study, we extracted data on publication year, study design, sample size, country, population characteristics, methods used for the diagnosis of MASLD and urolithiasis, outcomes of interest, matching and confounding factors included in multivariable regression analyses, and duration of follow-up (only for longitudinal studies). In the case of multiple publications of the same database, we included the most up-to-date or comprehensive information.

The overall quality of the studies included in the aggregate data meta-analysis was assessed using the Newcastle–Ottawa scale (NOS) by two independent authors (AM and GT). Any disparities in scoring were reviewed, and consensus was obtained following discussion. The NOS scale is a validated scale for non-randomized studies in meta-analyses, which uses a star system to assess the quality of a study in three domains: selection, comparability, and outcome/exposure. The NOS assigns a maximum of four stars for selection (or five stars in the case of cross-sectional studies), two for comparability, and three for outcome/exposure. We judged studies that received a score of at least 8 stars to be at low risk of bias, thus reflecting the highest quality.

### Data synthesis and analysis

The primary outcome measure of the meta-analysis was the presence of urolithiasis for cross-sectional studies or the risk of developing incident urolithiasis for longitudinal studies. The ORs (for cross-sectional studies) or HRs (for longitudinal studies) and their 95% CIs were considered as the effect size for all the eligible studies. When studies reported ORs/HRs with varying degrees of covariate adjustment, we extracted those that reflected the maximum extent of adjustment for potentially confounding variables. The adjusted ORs/HRs of all eligible studies were pooled, and an overall effect-size estimate was calculated using a random-effects model since high heterogeneity was expected for a meta-analysis of observational studies.

The statistical heterogeneity among studies was evaluated by the chi-square test and the *I*^2^-statistic, which estimates the percentage of variability across studies due to heterogeneity rather than chance alone. The proportion of heterogeneity accounted for by between-study variability was assessed using the *I*^2^-statistic and adjudicated to be significant if the *I*^2^ index was > 50% [18]. The possibility of publication bias was examined using the visual inspection of funnel plots and the Egger’s regression asymmetry test [19].

To explore the possible sources of (expected) high heterogeneity among the studies and to test the robustness of the observed associations, we performed subgroup analyses by study country (Asian vs. non-Asian countries), diagnostic methods used for identifying MASLD and urolithiasis (computed tomography vs. ultrasonography vs. survey questionnaires), and degrees of covariate adjustment (minimally vs. fully adjusted studies). We also tested for possible excessive influence of individual studies using a meta-analysis influence test that eliminated each included study at a time. Finally, we performed univariable meta-regression analyses to test the impact of age and sex on the effect size for the association between MASLD and urolithiasis.

All statistical tests were two-sided and used a significance level of *p* < 0.05. We used R version 4.3.3 (R Core Team 2023, R Foundation for Statistical Computing, Vienna, Austria. < https://www.R-project.org/ >) for all statistical analyses with the following packages: *meta* (version 7.0–0) and *metafor* (version 4.4–0).

## Results

### Literature search and characteristics of included studies

The PRISMA flow diagram of the meta-analysis is reported in Supplementary Fig. 1. After examining the titles and abstracts of the publications and excluding duplicates, we identified 13 potentially eligible studies from PubMed, Web of Science, and Scopus from the inception to March 31, 2024. We further excluded five studies because of unsatisfactory inclusion criteria (as reported in Supplementary Table 1). Consequently, we identified eight unique observational studies (7 cross-sectional studies and one prospective cohort study) for inclusion in the meta-analysis.

The main characteristics of these eight selected observational studies are reported in Table 1. Regarding the seven cross-sectional studies [20–26], the diagnoses of MASLD and urolithiasis were based on abdominal ultrasonography (*n* = 3 studies), non-contrast computed tomography (*n* = 3 studies), or survey questionnaires (*n* = 1 study). No studies were available on using liver biopsy to diagnose MASLD. Overall, these cross-sectional studies included 40,385 adult individuals (39.5% men; mean age 47.6 years; ~ 15% had a diagnosis of urolithiasis; ~ 22% had a diagnosis of MASLD). Five studies were conducted in Asia (China, South Korea, Iran, and Israel), one in the USA and one in Colombia. Two of these 7 studies obtained at least 8 stars on the NOS scale, thus reflecting a low risk of bias; two studies obtained 7 stars, and the remaining three studies obtained 6 stars. As shown in Table 1, the single published prospective cohort study was carried out in South Korea and included 208,578 adult individuals undergoing a health checkup examination who were followed for a median period of 6.6 years. The diagnosis of MASLD and urolithiasis was based on ultrasonography [27].

**Table 1:** Eligible cross-sectional (n = 7) and prospective studies (n = 1) examining the association between MASLD and risk of urolithiasis

Authors, Year (Ref.)	Study design	Study participants	MASLD diagnosis, % of cases^a^	Urolithiasis diagnosis, % of cases	Covariate adjustments	Study results	NOS
Cross-sectional studies							
Einollahi et al. (2013) [20]	Cross-sectional (retrospective analysis)	11,245 Iranian adult individuals (M/F: 5698/5547 mean age: NA mean BMI: NA % diabetes: NA)	Ultrasound,30% (*n* = 3341)	Ultrasound, 11% (*n* = 1193)	Sex	MASLD was associated with higher odds of urolithiasis (aOR 2.40, 95% CI 2.1–2.7)	7
Nam et al. (2016) [21]	Cross-sectional (retrospective analysis)	1381 South Korean adult individuals (M/F: 684/687 mean age: 55.8 years mean BMI: NA % diabetes: NA)	Non-contrast computed tomography, 18.2% (*n* = 251)	Non-contrast computed tomography, 11.6% (*n* = 160)	Age and sex	MASLD was associated with higher odds of urolithiasis (aOR 4.99, 95% CI 3.0–8.2)	6
Zeina et al. (2017) [22]	Cross-sectional (retrospective analysis)	508 Israeli adult individuals (M/F: 378/130 mean age: 47.5 years mean BMI: 29.5 kg/m^2^ % diabetes: 17.6%)	Non-contrast computed tomography, 15.7% (*n* = 80)	Non-contrast computed tomography, 83.9% (*n* = 421)	Age, sex, CT-measured visceral fat thickness and diabetes	MASLD was associated with higher odds of urolithiasis (aOR 2.52, 95% CI 1.02–6.2)	6
Wei et al. (2018) [23]	Population-based cross-sectional (retrospective analysis)	3719 Chinese adult male individuals (M/F: 3,719/0 mean age: 38 years mean BMI: 23.8 kg/m^2^ % diabetes: NA)	Ultrasound, 22.7% (*n* = 843)	Ultrasound, 4.0% (*n* = 149)	Age, BMI, education level, smoking, alcohol intake and physical activity	MASLD was associated with higher odds of urolithiasis (aOR, 1.35, 95% CI 1.01–1.81). In addition, increasing ultrasonographic severity of MASLD was associated with higher odds of urolithiasis (absent vs. mild vs. moderate vs. severe MASLD: aOR 0.78, 95% CI 0.51–1.21; aOR 1.23, 95% CI 0.75–2.03; aOR 2.11, 95% CI 1.08–4.14)	8
Decker et al. (2020) [24]	Population-based cross-sectional (NHANES III 1988–94) (retrospective analysis)	11,859 United States adult individuals (M/F: 5,450/6,409 mean age: 45 years mean BMI: NA % obesity: 26% % diabetes: 6%)	Ultrasound, 21.1% (*n* = 2498)	Survey questionnaires; percentage not available	Age, sex, ethnicity, smoking status, elevated serum liver enzymes, obesity, diabetes, hypertension and gout	MASLD was associated with higher odds of urolithiasis in women (aOR 1.65, 95% CI 1.17–2.32), but not in men (aOR 1.04, 95% CI 0.77–1.40)	8
Lubinus Badillo et al. (2020) [25]	Cross-sectional (retrospective analysis)	1,010 Colombian adult individuals (M/F: 500/510 mean age: NA mean BMI: NA % diabetes: NA)	Non-contrast computed tomography, 45.3% (*n* = 458)	Non-contrast computed tomography, 66% (*n* = 676)	Age and sex	MASLD was associated with higher odds of urolithiasis (aOR, 1.29, 95% CI 1.10–1.53). In addition, increasing ultrasonographic severity of MASLD was associated with higher odds of urolithiasis	6
Moftakhar et al. (2022) [26]	Population-based cross-sectional (retrospective analysis)	10,663 Iranian adult individuals (M/F: 4724/5939 mean age: 52 years mean BMI: NA % obesity: 18% % diabetes: 15%)	Survey questionnaires, 12.1% (*n* = 1285)	Survey questionnaires, 21.1% (*n* = 2251)	Age, sex, socioeconomic status, obesity, hypertension and diabetes	MASLD was associated with higher odds of urolithiasis (aOR, 2.21, 95% CI 1.93–2.52)	7
Prospective cohort studies							
Kim S et al. (2017) [27]	Prospective (retrospective analysis)	208,578 South Korean adult individuals who underwent a health check-up examination (M/F: 112,324/96,254 mean age: 38 years mean BMI: 24 kg/m^2^ % diabetes: 3% (n = 1593)). Median follow-up: 6.6 years (IQR 3.0–9.4 years)	Ultrasound, 24.4% (*n* = 50,964)	Ultrasound, *n* = 16,442 cases of incident urolithiasis over the follow-up (overall incidence rate, 1.6 per 100 person-years)	Age, center, year of screening exam, smoking, alcohol intake, physical activity, education level, BMI, hypertension, diabetes, HOMA-IR, C-reactive protein and uric acid	MASLD was associated with a higher risk of developing incident urolithiasis in men (aHR 1.17, 95% CI 1.06–1.30), but not in women (aHR 0.97, 95% CI 0.81–1.16)	8

*NA* not available, *aOR* adjusted odds ratio, *aHR* adjusted hazard ratio, *BMI* body mass index, *HOMA-IR* homeostasis model assessment-insulin resistance, *NOS* Newcastle–Ottawa Scale

^a^Originally labelled as NAFLD

### Cross-sectional studies on the association between MASLD and urolithiasis

**Fig. 1:**
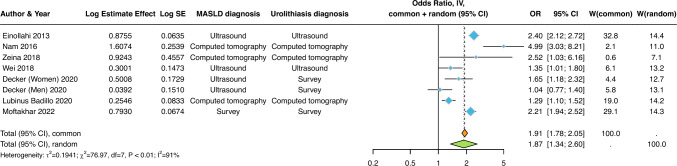
Forest plot and pooled estimates of the effect of MASLD on the risk of prevalent urolithiasis in the eligible cross-sectional studies

The distribution of cross-sectional studies (*n* = 7 studies involving 40,385 adult individuals from different countries) by estimate of the association between MASLD and the risk of prevalent urolithiasis is plotted in Fig. 1**.** We found that MASLD was significantly associated with a higher risk of prevalent urolithiasis (pooled random-effects OR 1.87, 95% CI 1.34–2.60; *I*^2^ = 91%). In most eligible studies, these results persisted when adjusted for age, sex, obesity, hypertension, type 2 diabetes, and other potential confounding factors (as specified in Table 1).

### Subgroup analyses

We undertook subgroup analyses to explore the possible sources of high heterogeneity across the cross-sectional studies. As shown in Supplementary Fig. 2, the association between MASLD and urolithiasis was stronger in Asian studies (*n* = 5 studies; pooled random-effects OR 2.38, 95% CI 1.58–3.57; *I*^2^ = 83%) than in non-Asian studies (*n* = 2 studies; random-effects OR 1.29, 95% CI 1.04–1.59; *I*^2^ = 51%). Conversely, the association between MASLD and urolithiasis was consistent when the comparison was stratified by different methods used for diagnosing MASLD (or urolithiasis) (Supplementary Fig. 3) or by the degree of covariate adjustment [pooled random-effects OR 2.42, 95% CI 1.15–5.09 for minimally adjusted studies (i.e., adjusted for age and sex), and pooled random-effects OR 1.59, 95% CI 1.12–2.17 for fully adjusted studies (i.e., adjusted for age, sex, obesity and other common metabolic risk factors)] (Supplementary Fig. 4).

### Sensitivity analyses and *meta*-regressions

A sensitivity analysis using the one-study remove (leave-one-out) approach to test the influence of each study on the overall effect size showed that eliminating each of the cross-sectional studies from the pooled primary analysis did not have any significant effect on the association between MASLD and urolithiasis (Supplementary Fig. 5). The results of univariable meta-regression analyses to examine the effect of potential moderator variables showed a significant positive association between age and urolithiasis (*p* = 0.011) (Supplementary Fig. 6). Conversely, meta-regression analysis did not show any significant effect of sex on the association between MASLD and urolithiasis (in cross-sectional studies) (*p* = 0.361) (Supplementary Fig. 7). As reported in Table 1, there were insufficient data to perform univariable meta-regression analyses for testing the effects of obesity and diabetes on the presence of urolithiasis.

### Cross-sectional studies on the association between more severe MASLD and urolithiasis

**Fig. 2:**
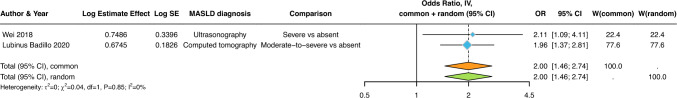
Forest plot and pooled estimates of the effect of ultrasonographic severity of MASLD on the risk of prevalent urolithiasis in the eligible cross-sectional studies

The distribution of cross-sectional studies by estimate of the association between the ultrasonographic severity of MASLD and the risk of prevalent urolithiasis is plotted in Fig. 2 [23, 25]. We found that more severe MASLD on ultrasonography was significantly associated with higher odds of urolithiasis (pooled random-effects OR 2.0, 95% CI 1.46–2.74; *I*^2^ = 0%). None of the eligible studies examined the association between blood-based biomarkers of liver fibrosis and the likelihood of urolithiasis.

### Longitudinal studies on the association between MALD and risk of developing urolithiasis

**Fig. 3:**
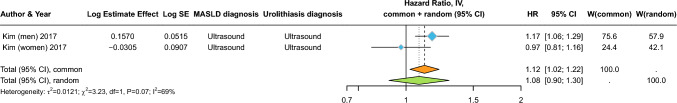
Forest plot and pooled estimates of the effect of MASLD on the risk of developing incident urolithiasis in the eligible cohort studies

The distribution of cohort studies by estimate of the association between MASLD and the risk of developing incident urolithiasis is plotted in Fig. 3. Only one cohort study from South Korea examined the association between MASLD and the risk of developing urolithiasis amongst 208,578 adult individuals undergoing health checkup examinations [27]. During a median follow-up of 6.6 years, there were 16,442 cases of incident urolithiasis. MASLD assessed by ultrasonography was significantly associated with a higher risk of incident urolithiasis in men (adjusted HR 1.17, 95% CI 1.06–1.30) but not in women (adjusted HR 0.97, 95% CI 0.81–1.16) after adjusting for common metabolic risk factors and other potential confounders. As shown in Fig. 2, when we plotted these two adjusted HRs in a forest plot analysis, MAFLD was not significantly associated with the risk of developing urolithiasis (pooled random-effects HR 1.08, 95% CI 0.90–1.30; *I*^*2*^ = 69%).

### Publication *bias*

**Fig. 4:**
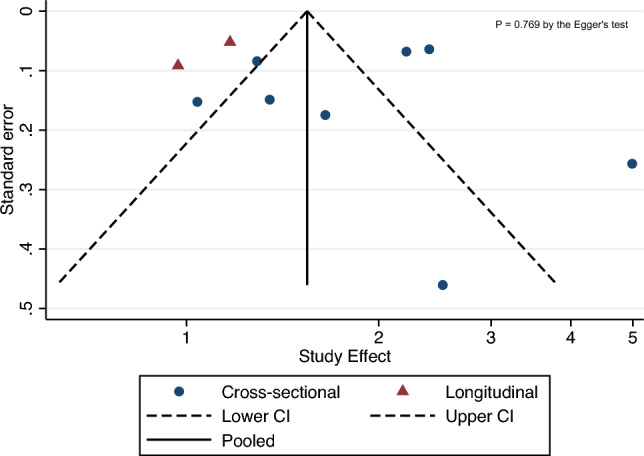
Funnel plot assessing the possibility of publication bias across the eligible studies (*n* = 8)

As shown in Fig. 4, the visual inspection and the Egger’s regression test (although less than ten studies were included) did not show any statistically significant asymmetry of the funnel plot for the included studies (*p* = 0.769), thus suggesting that the publication bias was low.

## Discussion

In this comprehensive and updated meta-analysis that included 8 observational studies (seven cross-sectional studies and one longitudinal cohort study) with aggregate data on ~ 250,000 adult individuals from different countries, we found that MASLD was significantly associated with an approximately two-fold higher prevalence of urolithiasis (*n* = 7 studies; pooled random-effects OR 1.87, 95% CI 1.34–2.60). This association was more robust in studies conducted in Asia than in non-Asian countries but remained unchanged when the comparison was stratified by different modalities for diagnosing MASLD or urolithiasis. Notably, this association remained significant in those studies where statistical analysis was adjusted for age, sex, ethnicity, obesity, type 2 diabetes, and other common metabolic risk factors. Moreover, the ultrasonographic severity of MASLD was significantly associated with higher odds of urolithiasis. Meta-regression analyses also showed a significant positive association between age and MASLD-related urolithiasis, suggesting that this association may be partly mediated by age population in different studies (thus explaining a part of the observed high heterogeneity among studies). Finally, the meta-analysis of data from the single Asian cohort study suggested that MAFLD was not associated with the risk of developing incident urolithiasis (pooled random-effects HR 1.08, 95% CI 0.90–1.30), although there was a significant interaction between sex and MASLD for the development of nephrolithiasis (MASLD being significantly associated with a higher risk of developing urolithiasis in men but not in women). Further well-designed prospective studies in Asian and non-Asian populations are needed to better understand whether MASLD may be a risk factor for incident urolithiasis Adequate consideration of sex differences and sex hormones/menopausal status in future clinical investigations will be also needed to fill current gaps and implement precision medicine. Further research is also required to elucidate whether the severity of MASLD (especially higher fibrosis stage, which is the strongest histologic predictor of hepatic and extrahepatic morbidity and mortality in MASLD [4, 28]) may adversely affect the risk of urolithiasis.

To our knowledge, this is the most updated and comprehensive meta-analysis assessing the association between MASLD and the risk of having or developing urolithiasis. Our findings corroborate and further expand the results of two previous meta-analyses published in 2018 [14, 15]. Unlike these two smaller meta-analyses, we included three large observational studies (published in 2020 and 2022) and performed separate analyses for cross-sectional and longitudinal studies (although only one Asian prospective cohort study was available so far), thus examining the possible distinct effects of MASLD on the prevalence and incidence of urolithiasis. Furthermore, our subgroup analyses and meta-regressions confirmed the robustness of the observed associations. Contrary to these previous meta-analyses [14, 15], we did not include “grey” literature, as we excluded unpublished studies and studies published outside widely available journals. Therefore, we did not include two cross-sectional studies from Colombia and Israel of very low quality that were published only as poster communications (as specified in our Supplementary material).

The findings of our meta-analysis may have some important clinical implications; more specifically, adult patients with MASLD may be screened for urolithiasis (especially in Asian people with MASLD) as these patients are at higher risk of urolithiasis. A recent Mendelian randomization study revealed no genetic evidence for a causal relationship between genetically predicted MASLD and the risk of urolithiasis [29]. Hence, routine screening for urolithiasis in patients with MASLD might seem poorly supported by current evidence. However, it should be noted that this Mendelian randomization study had some important limitations. First, the diagnosis of urolithiasis was based exclusively on International Classification of Disease (ICD) codes in the genome-wide association studies (GWAS) data (thus reducing the diagnostic accuracy in identifying MASLD). Second, the dataset included only individuals of European ancestry, so these results may not be generalizable to other ethnic populations [29].

There is intense debate about the independent contribution of steatotic/inflamed or fibrosing liver to the pathophysiology of urolithiasis in patients with MASLD. It is beyond the scope of this meta-analysis to discuss in detail the precise mechanisms whereby MASLD may contribute to the development of urolithiasis. Although further mechanistic studies are needed for establishing a cause-effect relationship between MASLD and the risk of urolithiasis, there is accumulating evidence of biological plausibility that MASLD may increase the risk of urolithiasis. MASLD and urolithiasis share multiple metabolic risk factors, such as obesity, dyslipidemia, type 2 diabetes and hyperuricemia/gout [30–32]. However, accumulating evidence indicates that MASLD may release multiple proinflammatory, lipotoxic and prooxidant mediators and exacerbate hepatic and systemic insulin resistance, i.e., a pathogenic factor in MASLD development, thus further contributing to the formation of renal stones. Insulin resistance may interfere with renal acid excretion and purine metabolism, causing unduly acidic urine pH and reduced urinary ammonium excretion [33, 34]. Insulin resistance may also act at the renal level by specific defects of basolateral transporters on the membrane and possibly by affecting the gatekeepers of the glomerular filtration barrier [34]. Preliminary evidence suggests that patients with MASLD also have a distinct urinary lithogenic risk profile characterized by reduced urinary magnesium and altered urinary ammonium excretion [35]. That said, it should be noted that none of the eligible studies had data on the chemical composition of renal stones, precluding any inference about the pathophysiological mechanisms potentially involved in the development of urolithiasis.

Our meta-analysis has some important limitations inherent to the design of the eligible studies. First, since the included studies had a retrospective cross-sectional (*n* = 7 studies) or prospective (only one study) design, it does not allow for the establishment of a cause-effect relationship between MASLD and urolithiasis. Second, although most studies adjusted the results for age, sex, smoking history and common shared metabolic comorbidities (such as obesity, type 2 diabetes and, in some cases, hyperuricemia/gout), the possibility of residual confounding by unmeasured factors (e.g., dietary habits) cannot be entirely excluded. Third, although we used a random-effects model, the interpretation of some meta-analysis results requires some caution because of the observed medium–high heterogeneity for the pooled primary analysis of studies and the relatively low quality of the studies, suggesting a medium–high risk of bias according to the NOS. Fourth, some studies diagnosed urolithiasis using survey questionnaires or ultrasonography, and only three studies used non-contrast computed tomography, which is the most accurate imaging method for detecting renal stones [36]; similarly, MASLD was diagnosed using imaging methods but not liver biopsy (i.e., the ‘gold standard’ for diagnosing and staging MASLD) [37, 38]. Therefore, it is hoped that more studies will use non-contrast computed tomography to diagnose urolithiasis in the future. Fifth, although all eligible studies have used the NAFLD nomenclature, for this meta-analysis, we have assumed that NAFLD and MASLD are synonymous, as the two fatty liver disease definitions share a very high level of overlap (> 95% of individuals with NAFLD meet the criteria for MASLD) [39, 40]. Finally, although a selective reporting bias of eligible studies could not be excluded, our comprehensive search has made it unlikely that any published studies were missed.

Notwithstanding these limitations, this meta-analysis also has important strengths. Our meta-analysis is the most comprehensive assessment of the association between MASLD and the risk of urolithiasis. The large number of individuals included (although the limited number of studies available so far) provided sufficient statistical power to quantify the magnitude of the association between MASLD and urolithiasis. Moreover, we used standardized risk estimates from all included studies to allow a consistent combination of estimates across eligible studies. Finally, the visual inspection of funnel plot did not reveal any significant asymmetry, thus suggesting that the risk of publication bias was low.

In conclusion, this updated meta-analysis of observational studies provides evidence for a significant association between MASLD and urolithiasis. This association was stronger in studies carried out in Asia than in non-Asian countries and remained statistically significant in those studies whose results were adjusted for age, sex, ethnicity, obesity, diabetes, and other potential confounders. There was a positive graded association between the ultrasonographic severity of MASLD and the presence of urolithiasis. It remains to be established whether individuals with MASLD also have a higher risk of developing incident urolithiasis. Future well-designed prospective studies in both Asian and non-Asian populations (possibly using vibration-controlled transient elastography or magnetic resonance-based techniques for diagnosing MASLD and using non-contrast computed tomography for detecting urolithiasis) are needed to further corroborate these findings and mechanistic studies to better decipher the complex mechanisms underpinning the association between MASLD and urolithiasis.

## Supplementary Information

Below is the link to the electronic supplementary material.

Supplementary file1 (PDF 504 KB)

## Data Availability

All supporting data of the meta-analysis are available within the article and in the online-only Supplementary Material.

## Abbreviations

NAFLD: Non-alcoholic fatty liver disease
MASLD: Metabolic dysfunction-associated steatotic liver disease
PRISMA: Preferred Reporting Items for Systematic Reviews and Meta-Analyses
NOS: Newcastle–Ottawa Quality Assessment Scale
OR: Odds ratio
HR: Hazard ratio
