# Supplementary material for: Association between metabolic dysfunction-associated steatotic liver disease and risk of urolithiasis: an updated systematic review and meta-analysis
Source: Intern Emerg Med. 2024 Jul 11;19(6):1745–55. doi: 10.1007/s11739-024-03705-5 (PMC11405432; doi:10.1007/s11739-024-03705-5)

# ONLINE-ONLY SUPPLEMENTARY MATERIAL

**Supplementary Table 1.** Observational studies excluded at the stage of eligibility according to the PRISMA flow diagram.

**Supplementary Figure 1**. The PRISMA flow diagram for search and selection processes of the meta-analysis.

**Supplementary Figure 2**. Forest plot and pooled estimates of MASLD on the risk of prevalent urolithiasis in the eligible cross-sectional studies, stratified by country.

**Supplementary Figure 3.** Forest plot and pooled estimates of MASLD on the risk of prevalent urolithiasis in the eligible cross-sectional studies, stratified by methods used for diagnosing MASLD.

**Supplementary Figure 4**. Forest plot and pooled estimates of MASLD on the risk of prevalent urolithiasis in the eligible cross-sectional studies, stratified by degree of covariate adjustment.

**Supplementary Figure 5.** One-study remove (leave-one-out) analysis to test the influence of each cross-sectional study on the overall effect size of the effect of MASLD on the risk of prevalent urolithiasis.

**Supplementary Figure 6**. Bubble plot with a fixed meta-regression line (in blue) about the pooled estimates of the effect of MASLD on the risk of prevalent urolithiasis by age in cross-sectional studies.

**Supplementary Figure 7**. Bubble plot with a fixed meta-regression line (in blue) about the pooled estimates of the effect of MASLD on the risk of prevalent urolithiasis by male sex in cross-sectional studies.

**Supplementary Table 1.** Observational studies *excluded* at the stage of eligibility according to the PRISMA flow diagram.

| Author, year | Study design | Main reason(s) for exclusion |
|---|---|---|
| Paz et al., 2015 | Cross-sectional study | Unsatisfactory study design (published as poster communication only) |
| Arias et al., 2018 | Cross-sectional study | Unsatisfactory study design (published as poster communication only) |
| Qin et al., 2019 | Cross-sectional study | Unsatisfactory study design (included only individuals with MASLD) |
| Qin et al., 2019 | Cross-sectional study | Unsatisfactory study design (included only individuals with MASLD) |
| Nguyen et al., 2022 | Cross-sectional study | Unsatisfactory study design (included only individuals with known or suspected urolithiasis) |

**References to the table**


1. Paz D GL. Association of renal stone (urolithiasis) with nonalcoholic fatty liver (NAFL). European Congress of Radiology (ECR). 2015; Poster No C-2056.
2. Arias CLF, Ramirez L, Ortiz O. Renal lithiasis and Fatty Liver Disease, is there any relationship? European Congress of Radiology (ECR). 2018; Poster No C-3221.
3. Qin S, Wang J, Zhou C, Zhang Y, Xu Y, Wang X, Wang S. The association between a non-invasive hepatic fibrosis score and urolithiasis among non-alcoholic fatty liver disease (NAFLD) patients in China: a cross-sectional study. BMJ Open. 2019;9(8):e027702.
4. Qin S, Wang J, Zhou C, Zhang Y, Xu Y, Wang X, Wang S. The severity of NAFLD is associated with the risk of urolithiasis. Br J Biomed Sci. 2019;76(2):53-58.
5. Nguyen DD, Bouhadana D, Wong P, Andonian S. What is the prevalence of hepatic steatosis on ultrasonography in patients followed for nephrolithiasis? Can Urol Assoc J. 2022;16(12):442-446.

**Supplementary Figure 1**. The PRISMA flow diagram for search and selection processes of the meta-analysis.

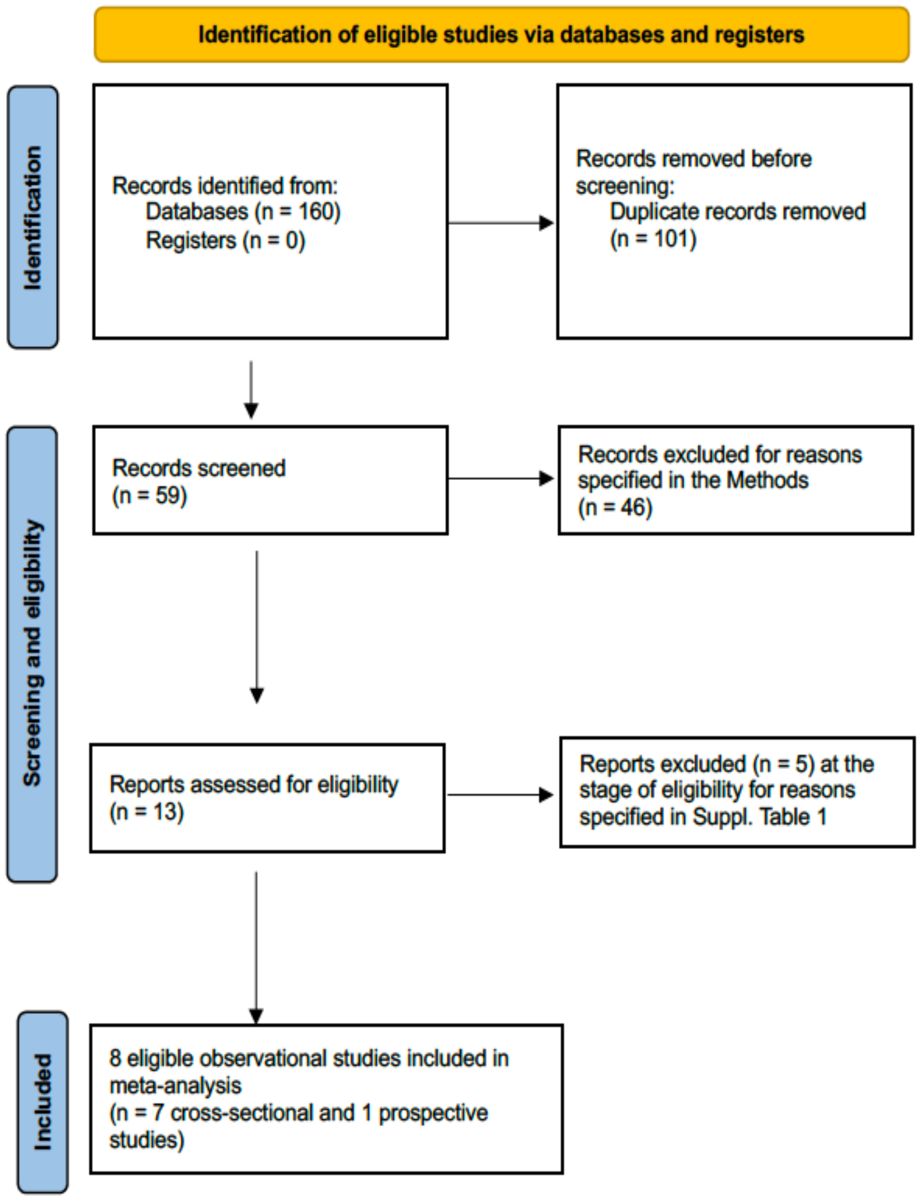

**Supplementary Figure 2**. Forest plot and pooled estimates of MASLD on the risk of prevalent urolithiasis in the eligible cross-sectional studies, stratified by country.

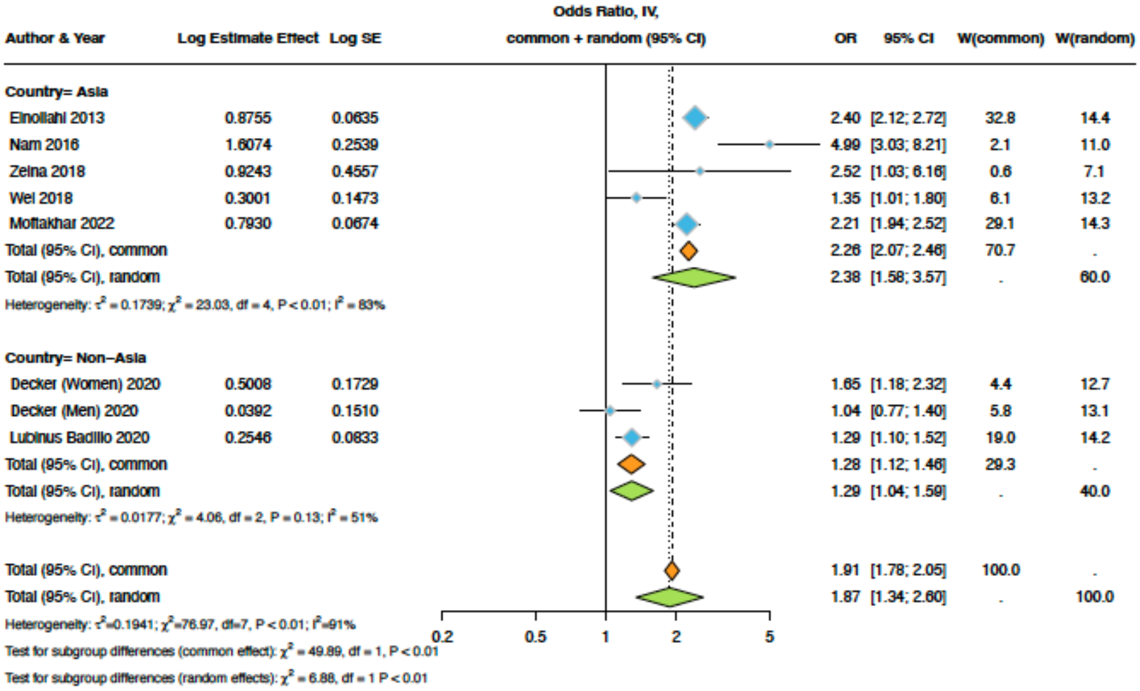

**Supplementary Figure 3.** Forest plot and pooled estimates of MASLD on the risk of prevalent urolithiasis in the eligible cross-sectional studies, stratified by methods used for diagnosing MASLD.

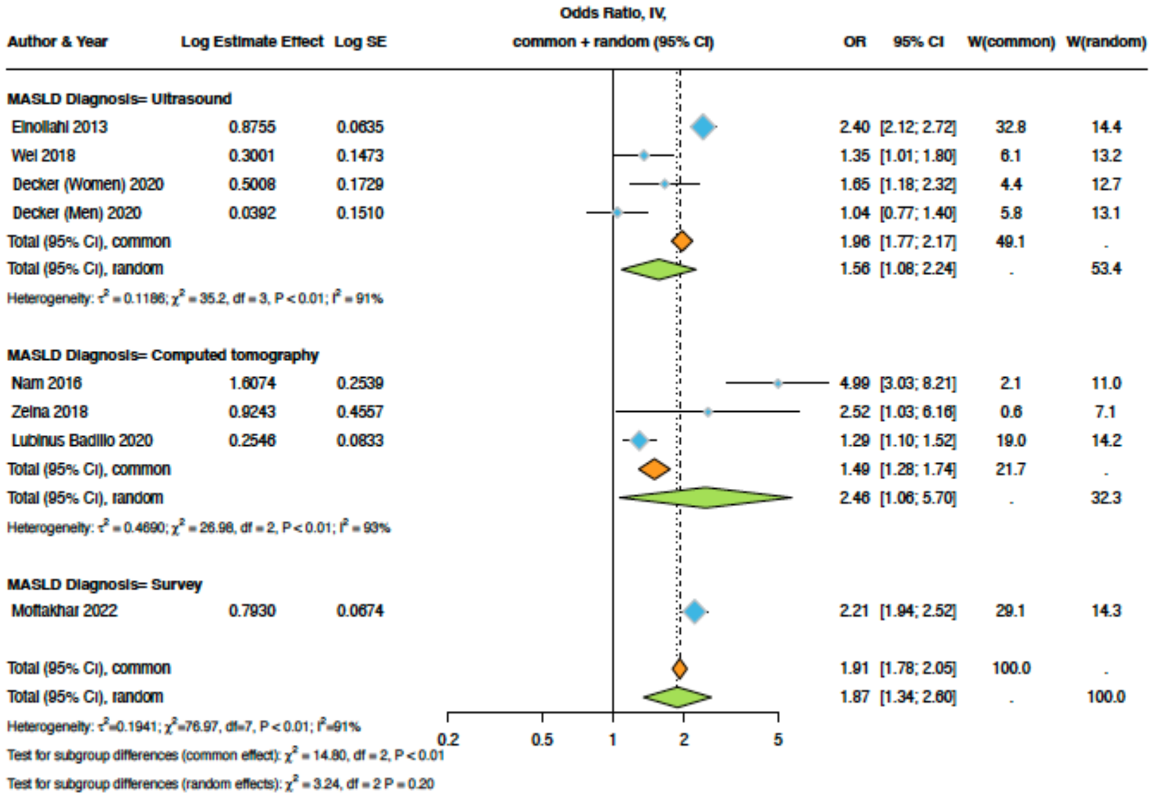

**Supplementary Figure 4**. Forest plot and pooled estimates of MASLD on the risk of prevalent urolithiasis in the eligible cross-sectional studies, stratified by degree of covariate adjustment.

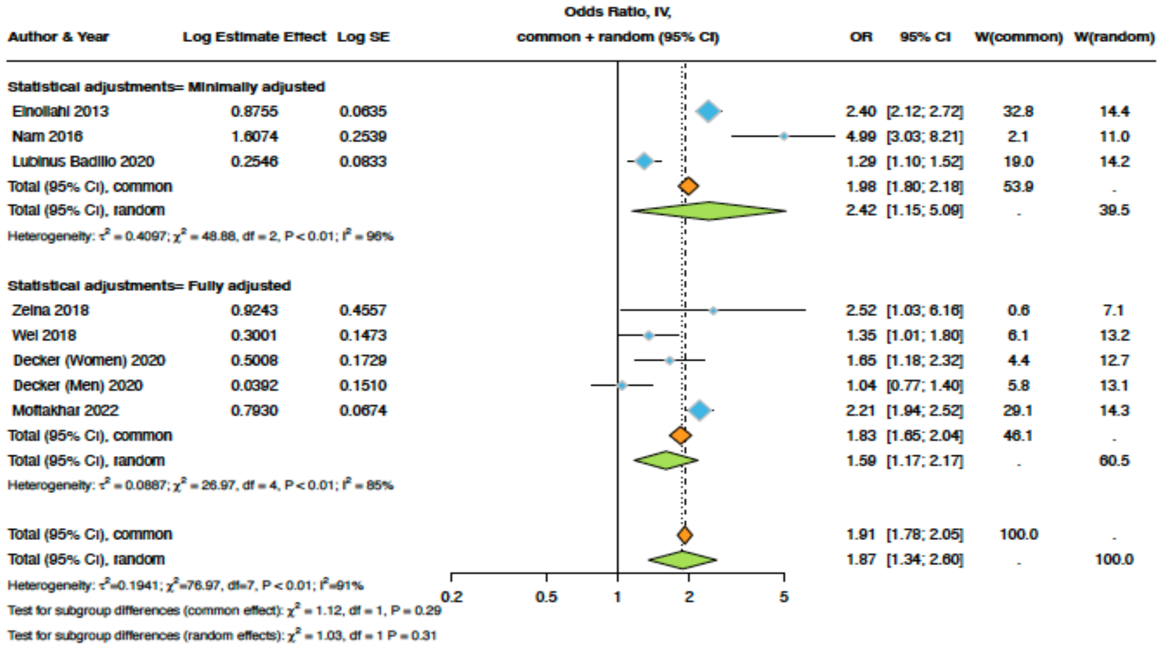

**Supplementary Figure 5.** One-study remove (leave-one-out) analysis to test the influence of each cross-sectional study on the overall effect size of the effect of MASLD on the risk of prevalent urolithiasis.

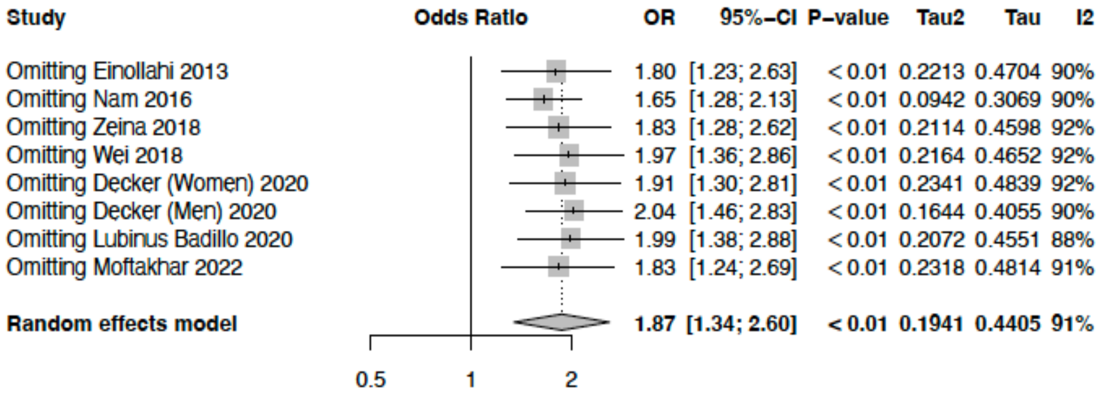

**Supplementary Figure 5.** One-study remove (leave-one-out) analysis to test the influence of each cross-sectional study on the overall effect size of the effect of MASLD on the risk of prevalent urolithiasis.

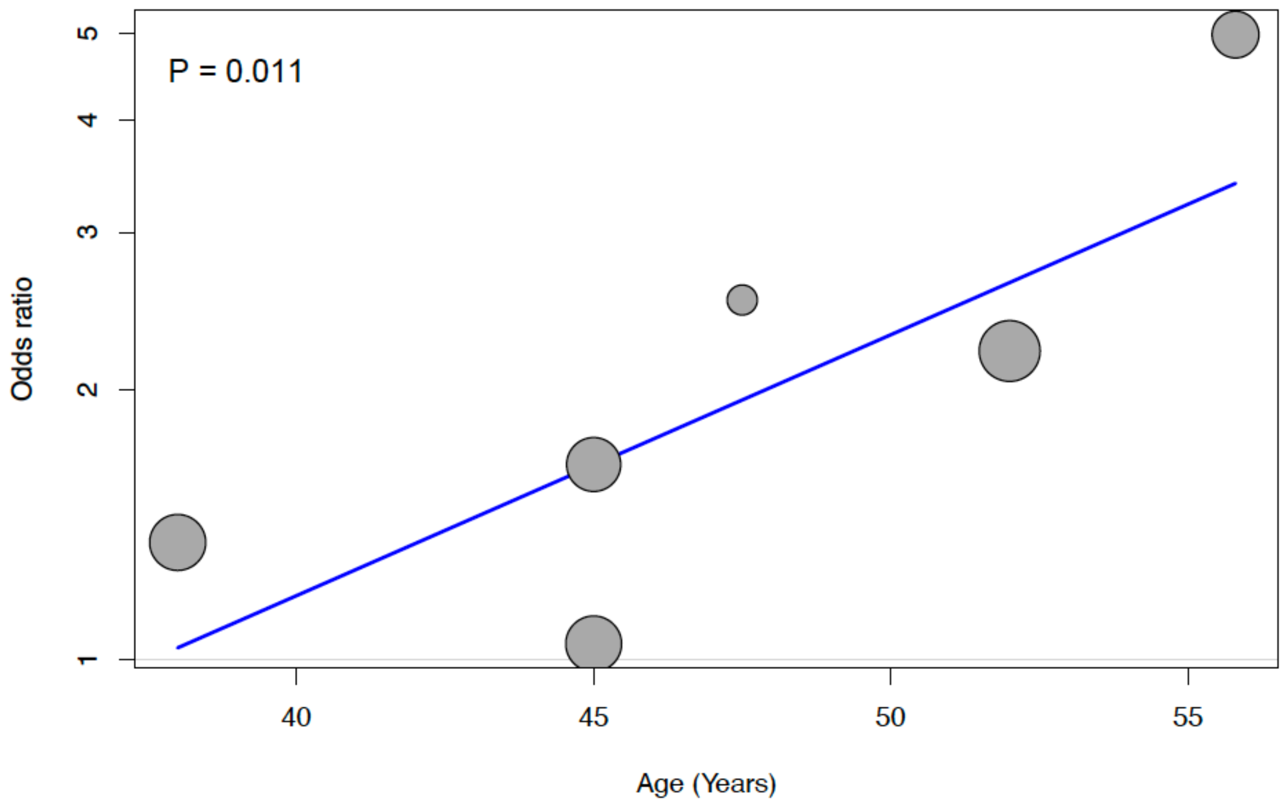

**Supplementary Figure 7**. Bubble plot with a fixed meta-regression line (in blue) about the pooled estimates of the effect of MASLD on the risk of prevalent urolithiasis by male sex in cross-sectional studies.

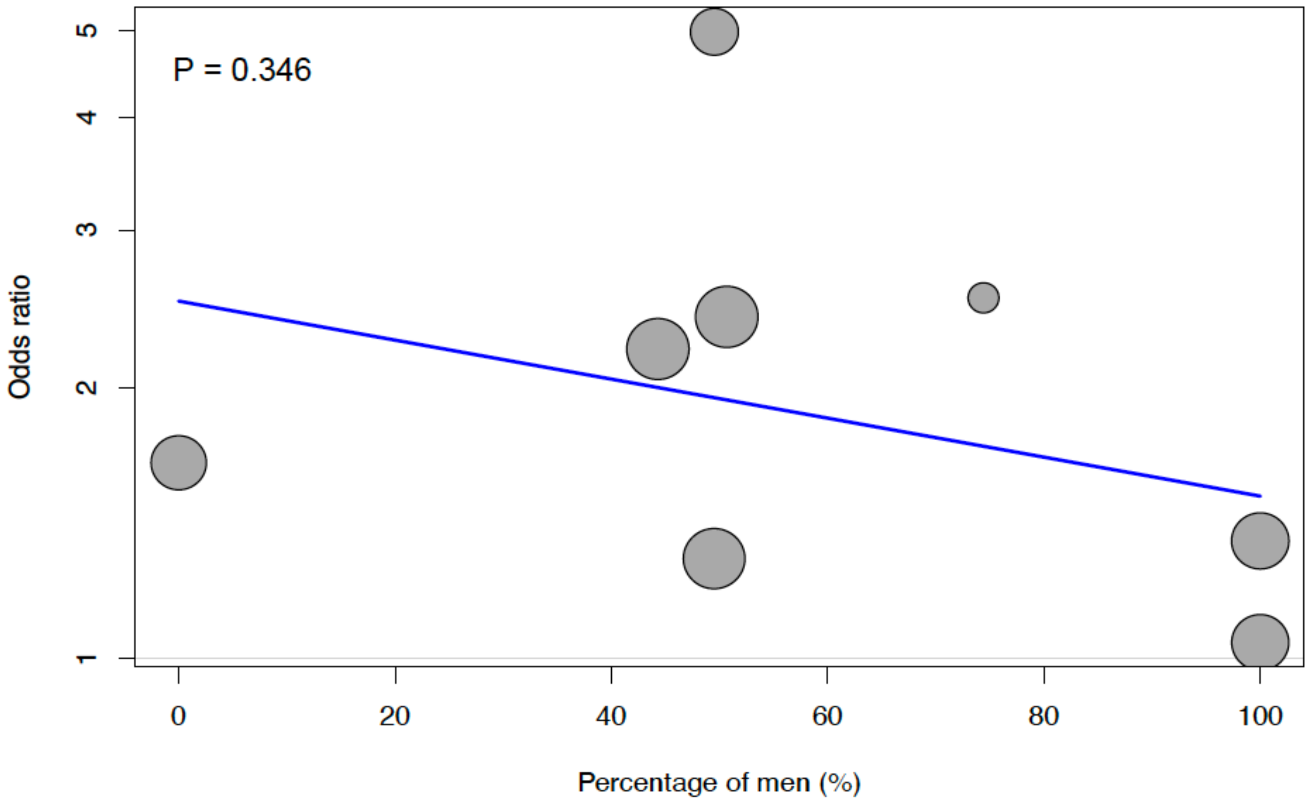

Supplement: Supplementary file 1 — Supplementary file1 (PDF 504 KB) [file 11739_2024_3705_MOESM1_ESM.pdf]
